# Decoding the Role of Platelets and Related MicroRNAs in Aging and Neurodegenerative Disorders

**DOI:** 10.3389/fnagi.2019.00151

**Published:** 2019-07-02

**Authors:** Yolanda Espinosa-Parrilla, Christian Gonzalez-Billault, Eduardo Fuentes, Ivan Palomo, Marcelo Alarcón

**Affiliations:** ^1^School of Medicine, Universidad de Magallanes, Punta Arenas, Chile; ^2^Laboratory of Molecular Medicine-LMM, Center for Education, Healthcare and Investigation-CADI, Universidad de Magallanes, Punta Arenas, Chile; ^3^Thematic Task Force on Healthy Aging, CUECH Research Network, Santiago, Chile; ^4^Laboratory of Cell and Neuronal Dynamics, Department of Biology, Faculty of Sciences, Universidad de Chile, Santiago, Chile; ^5^Geroscience Center for Brain Health and Metabolism GERO, Santiago, Chile; ^6^The Buck Institute for Research on Aging, Novato, CA, United States; ^7^Thrombosis Research Center, Department of Clinical Biochemistry and Immunohematology, Faculty of Health Sciences and Research Center for Aging, Universidad de Talca, Talca, Chile

**Keywords:** platelets, microRNAs, aging, Alzheimer disease, Parkinson disease, multiple sclerosis, Huntington disease and amyotrophic lateral sclerosis

## Abstract

Platelets are anucleate cells that circulate in blood and are essential components of the hemostatic system. During aging, platelet numbers decrease and their aggregation capacity is reduced. Platelet dysfunctions associated with aging can be linked to molecular alterations affecting several cellular systems that include cytoskeleton rearrangements, signal transduction, vesicular trafficking, and protein degradation. Age platelets may adopt a phenotype characterized by robust secretion of extracellular vesicles that could in turn account for about 70–90% of blood circulating vesicles. Interestingly these extracellular vesicles are loaded with messenger RNAs and microRNAs that may have a profound impact on protein physiology at the systems level. Age platelet dysfunction is also associated with accumulation of reactive oxygen species. Thereby understanding the mechanisms of aging in platelets as well as their age-dependent dysfunctions may be of interest when evaluating the contribution of aging to the onset of age-dependent pathologies, such as those affecting the nervous system. In this review we summarize the findings that link platelet dysfunctions to neurodegenerative diseases including Alzheimer’s Disease, Parkinson’s Disease, Multiple Sclerosis, Huntington’s Disease, and Amyotrophic Lateral Sclerosis. We discuss the role of platelets as drivers of protein dysfunctions observed in these pathologies, their association with aging and the potential clinical significance of platelets, and related miRNAs, as peripheral biomarkers for diagnosis and prognosis of neurodegenerative diseases.

## Introduction

Endothelial cells, coagulation factors and platelets essentially form the hemostatic system in blood. Platelets derive from the cytoplasm of megakaryocytes by fragmentation in bone marrow and circulate for 7–10 days in blood as disk-shaped anucleate particles (Machlus et al., 2014). Endothelial cells prevent the interaction of platelets with coagulation factors to avoid thrombosisunder normal conditions. However, disruption of the endothelium leads to extracellular matrix exposition that ultimately triggers a response known as primary hemostasis to repair tissue damage (Born and Cross, 1963).

Upon tissue damage, the glycoprotein IIb/IIIa complex (GP IIb/IIIa) facilitates platelet aggregation platelet-to-platelet interactions; allowing their binding to fibrinogen or von Willebrand factor (vWF) that bonds adjacent platelets. Morphologically, activated platelets radically change their shape from disks to become spiny spheres (Holinstat, 2017).

Platelets have two important types of granules: dense granules and alpha granules. The dense granules are loaded with proaggregatory factors such as 5-hydroxytryptamine serotonin, calcium and adenosine 5′-diphosphate (ADP). In platelet activation, these granules release their content to the open canalicular system to then be expelled by the platelet (Johnson et al., 1966). On the other hand, the alpha granules have many hemostatic proteins for example growth factors, e.g., platelet-derived growth factor, vWF and fibrinogen.

Once activated, platelets can recruit other platelets to the activation site: the release of proaggregatory substances, e.g., ADP, Thrombin and Calcium and the synthesis of prostanglandin. Thromboxane A2 (TXA2) is locally synthesized from arachidonic acid. These two mechanisms act concertedly to consolidate the initial recruitment by promoting the participation of other platelets in this site (Gryglewski and Ramwell, 1980). Additionally, when the platelets are activated, the phospholipids located on the inner side of the lipid bilayer are moved to the outer face of this lipid bilayer. Now this negatively charged surface provides binding sites for co-factors and coagulation enzymes, enhancing blood clot formation secondary hemostasis (Bevers and Zwaal, 1983).

## Platelets and Aging

The effect of age on the function of platelets is not yet fully elucidated Changes in platelet numbers and function have broadly been related to aging (Jones, 2016). It has been reported that platelet count remains relatively stable in people under 60 but decreases after that age, diminishing by around 8% about 20,000 platelets/μl (Segal and Moliterno, 2006).

Interestingly, platelet decline occurs even in the presence of an increase in the content of hematopoietic stem cells in aging suggesting that some of their functions are impaired (Hartsock et al., 1965). Such apparent paradox may be explained by defects observed in progenitor cells cycle and changes in the activity of some enzymes such as helicase (Flach et al., 2014).

It is worth noting that the increase in platelet activation is a reflection of the surrounding environment, for example endothelial activation increases platelet function and may end in thrombosis (Tomaiuolo et al., 2017). This situation is increased with age, due to an alteration in the mitochondrial function, among which is the mTOR pathway and alterations in the levels of fatty acids and glucose (Houtkooper et al., 2011).

Changes in the redox tone associated to aging contribute to the activation of platelets, and could be related with an increased production of reactive oxygen species (ROS) (Donato et al., 2013). There is a strong association between ROS levels and platelet activation leading to increased thrombotic diseases, diabetes and metabolic syndrome (Violi and Pignatelli, 2014; Bonomini et al., 2015). The activity of NADPH oxidase and superoxide dismutase, two enzymes responsible for producing ROS and H2O2, is significantly elevated in aged mice platelets (Drummond et al., 2011). Increased ROS are responsible for activating signaling pathways such as p38 MAP kinase that predisposes platelets to further activation resulting in an increased prothrombotic state (Herkert et al., 2002; Dayal et al., 2013).

Concomitantly, aging-dependent reduction of glutathione peroxidase and nitric oxide synthase activities contributes to increased platelets activations (Alexandru et al., 2008; Dayal et al., 2013).

With regard to platelet function aggregation there are several studies that indicate that it is altered in an age-dependent manner (Bastyr et al., 1990). A positive correlation between age and the ADP levels and beta-TBG a indication of platelet secretion *in vivo* was found when analyzing platelets from 40 healthy individuals 22 to 62-year-old with no clinical evidence of atherosclerotic vascular disease (Bastyr et al., 1990). Gleerup and Winther (1995) also found that platelet aggregation was significantly increased in a middle-aged group 41–72 years old compared with a young-aged group 21–30 years old, furthermore, in this last group vigorous exercise caused platelet agreeability to decrease, which was not observed in the middle-aged group.

Additionally, different authors have shown many alterations in platelets with respect to aging such as variations in the activities of protein serine/threonine kinases, signal transducers, ubiquitin-protein ligases and GTPases as well as in vesicle transport and cytoskeletal organization (Simon et al., 2014; Jones, 2016). Age-related variations in the expression of specific platelet receptors and platelet activators have also been reported and exemplified by a decrease in the number of receptors for PGI2 potent inhibitor of platelet function and high levels of TXA2 activator of platelet function in older individuals (Simon et al., 2014). All these assays come to show that platelets decrease in number but become hyperreactive in older adults.

## Platelet Micrornas and Extracellular Vesicles in Aging

The increase in platelet reactivity and aggregation observed in aging seems to be primarily driven by changes in platelet receptors and an increase in oxidative stress. However, differential regulation of gene expression by microRNAs (miRNAs) may also have an important role (Jones, 2016). miRNAs are small non-protein-coding RNAs involved in the post-transcriptional regulation of gene expression (Bartel, 2009). The functional miRNA molecule is the result of a maturation process that starts with the transcription of the miRNA gene into a primary miRNA transcript that is further processed by Drosha into a ∼70 nt hairpin miRNA precursor whose stem loop structure is recognized and cut by Dicer. The result of Dicer cleavage is a miRNA duplex formed by two molecules of mature miRNA of ∼20 nt (Krol et al., 2010). One of these mature miRNAs is then incorporated into RISC (Argonaute-containing miRNA induced silencing complex) that guides the interaction between the miRNA and its target messenger RNA (mRNA) resulting in gene expression inhibition either by degradation of the mRNA or by repression of the transcript translation (Bartel, 2009). Each mature miRNA may regulate hundreds of genes presenting a particular and characteristic spectrum of target genes (Friedman et al., 2009). Similarly, mRNAs may be regulated by several miRNAs, which is consistent with the idea that miRNAs function as controllers of complex gene networks that virtually include all known biological processes (Shalgi et al., 2007; Friedman et al., 2009). Involvement of miRNAs in disease has been largely reported in a plethora of human disorders (Hrdlickova et al., 2014; Vijayan and Reddy, 2016; Liang et al., 2018; Vijayan et al., 2018) with most research in the field being focused on neurodegenerative disorders (Weinberg and Wood, 2009; Sonntag, 2010) and more intensely on cancer (Lu et al., 2005; Meltzer, 2005; Elghoroury et al., 2018).

Numerous miRNAs are known to participate in the development and production of megakaryocytes and, ultimately, platelets (Edelstein and Bray, 2011; Dahiya et al., 2015; Sunderland et al., 2017). Platelets have fully functional miRNA machinery and display their own repertoire of miRNAs (Landry et al., 2009; Edelstein and Bray, 2011). In this regard, microarray expression profiling revealed a diverse and relatively rich population of miRNAs in human platelets being the most abundant miR-142-3p, miR-223, let-7a/c/i/b, miR-185, miR-126, miR-103, miR-320, miR-30c/b, miR-130a and miR-26 (Landry et al., 2009). Another microarray expression study also reported miR-142-3p, miR-223, miR-126, and miR-26 as the most expressed miRNAs in platelets (Simon et al., 2014). Additionally, a high-throughput sequencing-based analysis of human platelet miRNAs partially agreed with these two previous studies and revealed that more than 75% of the platelets expressed miRNAs were mainly from only 5 miRNA families that include let-7 the most abundant, representing 48%, miR-25, miR-103, miR-140, and miR-199 (Plé et al., 2012). These studies lead to the idea that platelets may represent one of the richest sources of human miRNAs. In addition, some of these and other miRNAs have been found to be important for either platelet production, reactivity, aggregation, secretion or adhesion; particularly miR-21, miR-34a, miR-96, miR-126, miR-146a, miR-150, miR-155, miR-200b, and miR-223; the last being exceptionally relevant for the transition from megakaryocytes to platelets and for platelet functioning (Edelstein and Bray, 2011; Dahiya et al., 2015; Fuentes et al., 2015). miR-223 has also been found deregulated in many inflammatory conditions (Edelstein and Bray, 2011; Gatsiou et al., 2012; Dahiya et al., 2015; Fuentes et al., 2015) and has further been described as a neuroprotective molecule due to its capacity to control responses to neuronal injury by controlling the functional expression in the brain of the *N*-methyl-D-aspartate receptor subunit NR2B and the ionotropic AMPA glutamate receptor 2 GluR2 (Nagy et al., 2004).

Interestingly, several of these platelet miRNAs have been included in a group of miRNAs named geromiRs because of their involvement in aging-related processes (Ugalde et al., 2011). Of special interest are miR-146a and miR-155 that have been specifically associated with inflammatory processes mediating brain aging (Olivieri et al., 2013). Additionally, the expression of certain miRNAs in platelets is found to be coordinated with platelet reactivity and with pathological states that may be related to aging (Edelstein and Bray, 2011; Pienimaeki-Roemer et al., 2017; Sunderland et al., 2017). According to this, in a study performed on 154 healthy subjects, fifteen platelet miRNAs were found to be differentially controlled by age, eleven of these miRNAs decreased with older age and, conversely, their respective target mRNAs increased their expression (Simon et al., 2014).

Senescent and/or activated platelets are widely found in vascular and neurological disorders and are known to induce the liberation of platelet extracellular vesicles (EVs) (Aatonen et al., 2012; Pienimaeki-Roemer et al., 2015, 2017) making up about 70–90% of all circulating vesicles in the blood (Hunter et al., 2008; Flaumenhaft et al., 2009; Gatsiou et al., 2012; Dahiya et al., 2015). EVs are an heterogeneous group of particles that are enriched in miRNAs, which are largely protected from degradation when carried by EVs, and may exert important regulatory functions in platelet activation pathways associated with platelet hyperactivity as well as functioning as mediators in the communication between platelets and other cells (Edelstein and Bray, 2011; Gatsiou et al., 2012; Laffont et al., 2013; Pienimaeki-Roemer et al., 2017). This is illustrated by miR-223, one of the most abundant miRNAs found in platelet EVs, which was shown to be transferred from platelet EVs to endothelial cells regulating pro-apoptotic gene pathways (Laffont et al., 2013). Also several miRNAs enriched in platelet EVs have been shown to be deregulated in the blood and/or brain of patients diagnosed with a neurodegenerative disease (Pienimaeki-Roemer et al., 2017). It is the case, for example, of the geromiR miR-146, which is carried by platelet EVs and is deregulated in the blood of patients with Parkinson disease (PD) and in the blood, hippocampus and frontal cortex of patients with Alzheimer disease (AD) (Table 1). In this sense, increasing evidence suggests that senescence-associated EVs, which are EVs secreted by senescent cells, could be novel senescence-associated secretory phenotype factors with unique characteristics that could participate on tuning the phenotype of recipient cells, through their ability to function as intercellular communicators (Willeit et al., 2013; Kadota et al., 2018; Takasugi, 2018).

**Table 1:** Platelet-related and Platelet-EVs-related miRNA families more frequently involved in neurodegenerative disorders.

miRNA	Disorder	Evidence for involvement of miRNAs in neurodegenerative disorders	References
miR-15a/b	AD	Down-regulated in the plasma of patients	Wang et al., 2011
	AD	Hyperphosphorylation of Tau protein through up-regulation of ERK kinases	Hébert et al., 2010
	MS	Potential informative biomarker to distinguish relapsing-remitting from progressive MS	Ebrahimkhani et al., 2017
	MS	Predicted regulation of *FGF2* and *KIF1B*	Fenoglio et al., 2012
	HD	Up-regulated in the frontal cortex of patients	Martí et al., 2010
miR-16	AD	Regulation of *APP*	Maciotta Rolandin et al., 2013
	PD	Up-regulated in the blood of levodopa treated patients	Margis et al., 2011
	MS	Up-regulated in the blood of patients	Keller et al., 2014
	HD	Up-regulated in the striatum and frontal cortex of HD patients	Martí et al., 2010
miR-20a/b	AD	Regulation of *APP*	Hébert et al., 2009; Maciotta Rolandin et al., 2013
	MS	Down-regulated in the blood of patients	Keller et al., 2014
	MS	Up-regulated in the plasma of patients	Martí et al., 2010
	HD	Up-regulated in the frontal cortex of patients	Siegel et al., 2012
miR-29a/b	AD	De-regulated in the brain of patients	Hébert et al., 2008
	PD	Down-regulated in the blood of patients	Margis et al., 2011
	PD	Up-regulated in the blood of levodopa treated patients. Predicted regulation of *PARK7*, *GPR37*, *CDC42*, *BACE1*, and *BCL2*	Maciotta Rolandin et al., 2013; Serafin et al., 2015
	HD	Up-regulated in the Brodmann’s area 4 of patients	Johnson et al., 2008
	HD	Down-regulated in the Brodmann’s area 4 of HD patients	Packer et al., 2008
miR-30b/c/e	AD	Up-regulated in the cerebrospinal fluid of patients	Cogswell et al., 2008
	AD	Up-regulated in circulating exosomes of patients	Tan et al., 2014; Cheng et al., 2015
	AD	Deregulated in several areas of the brain of patients	Cogswell et al., 2008
	PD	Up-regulated in the blood of levodopa treated patients. Predicted regulation of *LRRK2* and *BCL2*	Margis et al., 2011; Serafin et al., 2015
	MS	Potential informative biomarker to distinguish relapsing-remitting from progressive MS	Ebrahimkhani et al., 2017
miR-34a/b/c	AD	Up-regulated in blood mononuclear cells of patients	Schipper et al., 2007; Burgos et al., 2014; Kiko et al., 2014
	AD	Up-regulated in the hippocampus and frontal cortex of patients	Cogswell et al., 2008; Banzhaf-Strathmann et al., 2014; Müller et al., 2014
	AD	Up-regulated in the serum of patients	Bhatnagar et al., 2014
	AD	Affecting the clearance of Tau from the circulation through repressing *SIRT1*	Schonrock and Götz, 2012
	PD	Down-regulated in amygdala, frontal cortex and cerebellum patients. Regulation of *PARK7* and *PRKN*	Miñones-Moyano et al., 2011
	HD	Up-regulation in the plasma of patients	Gaughwin et al., 2011
miR-146a/b	AD	Up-regulated in the hippocampus and frontal cortex of patients	Cogswell et al., 2008; Müller et al., 2014
	AD	Down-regulated in the plasma of patients	Kiko et al., 2014
	AD	Deregulated in the cerebrospinal fluid of patients	Cogswell et al., 2008; Alexandrov et al., 2012
	AD	Regulation of *CFH* and *TLR4*	Pienimaeki-Roemer et al., 2017
	PD	Down-regulated in the blood of patients	Caggiu et al., 2018
	ALS	Up-regulated in the spinal cord of patients. Regulation of *NFL*	De Felice et al., 2012
	ALS	Up-regulated in CD14+ CD16- monocytes of patients	Butovsky et al., 2012
miR-155	AD	Regulation of *APP*	Maciotta Rolandin et al., 2013
	AD	Up-regulated in the cerebrospinal fluid of patients	Alexandrov et al., 2012
	PD	Up-regulated in the blood of patients. Promising as target for anti-inflammatory therapy	Caggiu et al., 2018
	MS	Up-regulated in the brain and plasma of patients. Regulation of *CD47*	Jagot and Davoust, 2016
	ALS	Up-regulated in the spinal cord of patients. Potential therapeutic target	Koval et al., 2013
	ALS	Up-regulated in CD14+ CD16- monocytes of patients	Butovsky et al., 2012

*FGF2, fibroblast growth factor-2; *KIF1B*, Kinesin family member 1BAPP; *APP*, amyloid precursor protein; *PARK7*, protein deglycase DJ1; *GPR37*, G Protein-Coupled Receptor 37; *CDC42*, cell division control protein 42 homolog; *BACE1*,β-secretase; *BCL2*, apoptosis regulator; *SIRT1*, sirtuin; *PRKN*, parkin; *CHF*, complement factor H; *TLR4*, Toll-like receptor 4; *NFL*, low MW neurofilament; *CD47*, cluster of differentiation 47*.

## Platelets and Neurodegenerative Diseases

Even though the most important function of platelets is to prevent bleeding (Alarcon et al., 2015), they also play an important function in pathological conditions, as for example neurological and neurodegenerative diseases, including PD (Lim et al., 2009), Schizophrenia (Asor and Ben-Shachar, 2012), and AD (Ciabattoni et al., 2007). It is also important noting that platelets show high expression of several proteins associated with the development of AD, such as the APP amyloid precursor protein (Bush et al., 1990) and tau protein (Mukaetova-Ladinska et al., 2013). Additionally, platelets express enzymes involved in protein modifications such as Glycogen synthase kinase 3 β (GSK-3β) (Li et al., 2008), α, β, and γ secretases (Smith et al., 2009). Of note, platelets have been compared with neurons because they have many biochemical similarities (Talib et al., 2012), as it is the storage and release of neurotransmitters from platelets such as serotonin, glutamate and dopamine (Cupello et al., 2005; Rainesalo et al., 2005) and the expression of neuron-related proteins such as NMDA receptors (Kalev-Zylinska et al., 2014). Together this makes it interesting to consider the contribution of platelets to the hallmarks of neurodegeneration.

Neurodegenerative diseases affect cells primarily neurons in the central nervous system (CNS) including the brain, spinal cord, the optic and olfactory nerves but some times may also affect the peripheral system (PNS). Many of the neurodegenerative diseases with no cure are associated with different symptoms such as progressive degeneration and/or death of nerve cells producing a lot of problems related to movement, e.g., ataxias, and/or mental functioning, e.g., cognitive impairment and dementias. Dementias are responsible for the highest percentage of neurodegenerative diseases, contributing to AD, which represents around of the 60–70% of dementia cases (Selkoe, 2001).

## Platelets and Alzheimer’s Disease

Alzheimer disease is a chronic progressive neurodegenerative disorder characterized by memory decline and several alterations at the cognitive level. It is the most common cause of dementia in the elderly being calculated that 26.6 million people worldwide suffer from AD, and whose prevalence is estimated to quadruple by 2050 (Qiu et al., 2009).

There are several forms of AD, although the patients who develop clinical symptoms older than 65 years are the great majority (late onset AD, LOAD; 95%), the rest (5%) of patients have an earlier onset of the disease (early-onset AD) (Plagg and Humpel, 2015). The early onset AD is related to the existence of rare autosomal dominant forms of AD, which manifest as early onset AD, although most of these patients do not present a pattern of autosomal clear inheritance. However, genetic predisposition is very important, even in patients with late-onset AD, which estimated heritability is 60–80% (Gatz et al., 2006).

The two major hallmarks of the disease in patients with AD are the presence of senile plaques and neurofibrillary tangles in the brain, which are related to vascular dysfunction, inflammation and neurological damage including loss of synapses and glial and cholinergic degeneration (Polanco et al., 2018).

Another element that should be highlighted is the relationship between AD and oxidative stress, which has been shown to participate in the development of AD and vascular dementia (Luca et al., 2015).

Swerdlow was the first to associate the hypothesis of mitochondrial dysfunction with the early pathological events that occurred in AD (Swerdlow and Khan, 2004). He pointed out that the increase and accumulation of the amyloid β (Aβ) peptide produces an alteration in the mitochondria that leads to an increase in oxidative stress and neuroinflammation including apoptosis that leads to the development of AD (Hauptmann et al., 2006). Specifically, deposits of the Aβ peptide have been found in the mitochondria causing an alteration in the mitochondrial respiration since it affects the enzymatic complexes III and IV. This produces a decrease in the production of ATP and increase of the production of ROS (Rhein et al., 2009; Swerdlow et al., 2010). The above results in the opening of the mitochondrial permeability transition pore (mPTP), increasing oxidative stress and apoptosis, inducing the liberation of cytochrome C and producing damage and mutation of mitochondrial DNA (Hauptmann et al., 2006; Lakatos et al., 2010; Calkins et al., 2011). All these processes enhance neurodeneration (Manczak et al., 2011).

The activity of several enzymes that regulate oxidative stress such as cytochrome oxidase and mitochondrial pyruvate dehydrogenase is affected in the brain of AD patients resulting in a diminished barrier against oxidative stress (Swerdlow, 2011). *In vitro* tests have been able to demonstrate that the Aβ peptide could increase the levels of lipid peroxides and hydrogen peroxide and in this way it would relate to AD and vascular dementia (Gustaw-Rothenberg et al., 2010).

Even in cultures of hippocampal neurons the soluble Aβ peptide induced high levels of ROS producing a great synaptic damage and neuronal loss, in a way that could explain some of the toxicity mechanisms of the peptide (De Felice et al., 2007).

Moreover some research shows oxidative stress as responsible for the generation of this peptide, as an example transgenic mice over expressing the APP, and whose antioxidant system is altered, showed a significant increase in the deposit of this peptide in the brain (Li et al., 2004).

Also oxidative stress is capable of altering the intracellular location of the β-secretase the enzyme responsible for processing the β- APP, promoting the amyloidogenic processing of the APP, which consequently increases the Aβ peptide (Tan et al., 2013). In the brains of patients with AD characteristic effects of oxidative stress, i.e., oxidation of lipids, protein and DNA damage have been shown (Butterfield et al., 1999) and increase in ROS levels were observed (Christen, 2000).

There are studies that have demonstrated that the Aβ peptide 1–40 (Aβ_40)_ possesses a capacity to generate free radicals that are very important in AD pathogenesis, because in position 35 it possesses a methionine which is classified as an active redox amino acid vital in the neurotoxicity of peptide (Hensley et al., 1994). It has been demonstrated that by substituting methionine for a noreleucine exchange of a sulfide group for a methylene, the neurotoxic properties of this peptide are considerably reduced (Varadarajan et al., 1999).

It is also important to note that this peptide is capable of generating ROS in hippocampal (Varadarajan et al., 2000) and cortical synaptosomes (Kanski et al., 2002) and significantly increases the levels of carboxylated proteins in cortical synaptosomes (Ansari et al., 2006). These same results were corroborated in cortical synaptosomes from knockout mice in APOE, where it was shown that the Aβ_40_ peptide produces oxidation and peroxidation of proteins and lipids (Keller et al., 2000; Lauderback et al., 2001).

Other qualities of this peptide that promote AD are that prior to neuronal damage the Aβ_40_ peptide significantly produces a considerable reduction in the Na^+^/K^+^-ATPase activity in hippocampal neurons of rats, which would favor neuronal death (Mark et al., 1995).

Glutamine synthase is another affected enzyme that loses its activity when incubated with this peptide in hippocampal neurons, which exacerbates the neurotoxic capabilities of Aβ_40_ peptide (Aksenov et al., 1995; Harris et al., 1995). Similarly, a loss of function and activity of glutamine synthase has been observed in AD brains (Smith et al., 1991; Butterfield et al., 1997).

### Platelets and Amyloid β-Peptide

The abnormal accumulation of the Aβ gives rise to senile plaques, its structure is in the form of β-plated sheet fibrils in cerebral arteries and capillaries nervous tissues. Cerebral amyloid angiopathy (CAA) is a disorder characterized by deposits of Aβ_40_ in cerebral arteries and capillaries (Pezzini et al., 2009), with an estimated prevalence of 90–98% in AD patients. Also the CAA is present in 30% of individuals without dementia over 60 years old (Weller et al., 2009). This disease increases the risk of haemorrhagic stroke, dementia and contributes to neurodegeneration and thus to cognitive decline, being a key factor in the etiology of AD (Jellinger and Attems, 2007). It is very imperative to note that the brains of CAA patients show several alterations in cerebrovascular tissues like endothelial cell alteration, i.e., elevated levels of adhesion molecules: VCAM-1 (vascular cell adhesion protein 1), ICAM-1 (Intercellular Adhesion Molecule 1), *E*-selectin (Endothelial Leukocyte Adhesion Molecule-1), (Zuliani et al., 2008), inflammatory interleukin (IL-1β, IL-6, IL-8) and other molecules such as TNFα (tumor necrosis factor alpha), TGFβ (transforming growth factor beta), MCP-1 (monocyte chemoattractant protein-1) and matrix metalloproteases (Grammas, 2011). All these alterations lead to a proinflammatory state in the brain neuroinflammation, for example the IL-1β and TNFα increase the blood–brain barrier permeability and tight junctions (Steinman, 2013) generating neuronal degeneration, and memory dysfunction.

Aβ peptide is derivative from APP, which is a large type I transmembrane protein (Masters et al., 1985). APP is present in brain and in cells that circulate peripherally such as lymphocytes, monocytes and interestingly, it is also highly expressed in platelets (Bush et al., 1990). Many APP isoforms arise from alternative splicing but the three most common isoforms are APP695, APP751 and APP770, where APP695 is highly expressed in neurons while APP751 and APP770 are expressed in platelets (Li et al., 1999). Human platelets have high levels of APP and are thought to contribute to more than 90% of circulating APP (Li et al., 1994). Regulation of APP alternative splicing is therefore important to determine tissue specificity of APP isoforms, a process that is at least partially mediated by miRNAs (Smith et al., 2011). This has been proven specifically for the neural specific miRNA the miR-124 and its direct target PTBP1 (polypyrimidine tract binding protein 1) (Li et al., 1994; Makeyev et al., 2007). PTBP1 is highly implicated in the regulation of alternative splicing in the brain and its expression levels are tightly related to both, neuronal APP splicing and miR-124 expression (Li et al., 1994; Makeyev et al., 2007). The expression of miR-124 is down-regulated in patients with AD (Li et al., 1994; Lukiw, 2007), which could result in abnormal neuronal splicing of APP and, consequently, affect β-amyloid peptide production (Niwa et al., 2008; Smith et al., 2011). These findings provide new perspectives into the physiological and pathological role for miRNA-mediated regulation of APP in AD.

Amyloid precursor protein is post-translationally processed in two different ways depending on the secretases involved in its cleavage, one pathway leads to amyloid plaque formation amyloidogenic, while the other does not produce peptide aggregation in pathological deposits non-amyloidogenic (Selkoe and Hardy, 2016). In the non-amyloidogenic pathway, the extracellular domain of APP is cleaved by an enzyme called the α-secretase generating the soluble amyloid precursor protein α (sAPPα) and C-terminal fragment α (CTFα) retained in the membrane, where it is acted upon by the γ-secretase complex including different enzymes such as Anterior Pharynx defective 1, Nicastrin, Presenilin enhancer 2, Presenilin 1 and or Presenilin 2, generating two fragments that are the amyloid precursor protein intracellular domain (AICD) and a soluble N-terminal fragments p3 (Shoji et al., 1992; Chang and Suh, 2010). Meanwhile, APP is cut by β-secretase *BACE1* to produce a soluble amyloid precursor protein β (sAPPβ) and a C-terminal fragment β (CTFβ) also retained in the membrane, which is subsequently processed close to the N-terminal to generate CTFβ. Finally, the CTFβ is cleaved byγ-secretase complex producing the Aβ peptide that is larger than p3 and AICD (Zhang et al., 2012). The Aβ peptide may vary in size from 38 to 43 amino acids, depending on the cutting activity of theγ-secretase where it mainly produces two isoforms where the Aβ_40_ is the most abundant ∼80–90%, followed by Aβ_42_ ∼5–10%. This last isoform is more toxic and hydrophobic and is capable of being added in oligomers and fibrils to form the extracellular plaques that are deposited in the brain (Selkoe, 2001). The most important circulating peptide is Aβ_40_ over 95%, and in AD contributes to the formation of perivascular amyloid plaques (Li et al., 1994; Chen et al., 1995; Herzig et al., 2004; Fryer and Holtzman, 2005).

Platelets express all the necessary enzymes for Aβ_40_ production α, β, and γ-secretases and release all the segments of APP: sAPPα, sAPPβ and Aβ that may also be stored into alpha granules (Li et al., 1998). The processing of APP may occur at two different sites, either in the intracellular organelles secretory pathway or on the platelet surface (Li et al., 1998; Evin et al., 2003) being released as exocytosis products of the increase of intra-platelet calcium (Ca^2+^) levels by two agonists: Thrombin and Collagen (Bush et al., 1990; Evin et al., 2003). It is worth to remark that there have been high levels thrombin in senile plaques in patients with AD (Akiyama et al., 1992). Increased protein kinase C (PKC) activity dependent on phosphatidylinositol 3-kinase (PI3K) has also been reported (Barry et al., 1999; Skovronsky et al., 2001) in platelets releasing Aβ_40_ that ultimately leads to calpain activation and the consequent increase of Aβ secretion by the platelets (Chen et al., 2000; Getz, 2012). Indeed calpain activation is involved in p35 protein processing that leads to increased Cdk5 activation involved in AD (Contreras-Vallejos et al., 2012).

Aβ peptide released from activated human platelets contributes to vascular amyloid deposits, as previous studies have shown that the Aβ infiltration induces a cellular replacement in the vasculature, specifically in media and adventitia layers, leading to thinner vessel wall damage (Mandybur, 1986). Additionally, a decrease in the expression of tight junction proteins claudin-1 and claudin-5 and increased matrix metalloproteases 2 and 9 production enhance vessel wall damage (Hartz et al., 2012). All of these changes induce blood vessel rupture resulting in a intracerebral hemorrhage with a decrease in blood flow and a suspension of oxygen supply to the brain, possibly inducing neuronal loss involved in dementia (Song et al., 2014). In addition, endothelial cells present in the vasculature express the receptor for advanced glycation end products (RAGE) that has been previously linked to neurodegeneration in a mechanism involving the exposure of endothelial cells to Aβ_40_ peptide, increased levels of inflammatory cytokines (Interleukin-6, IL-6; Interleukin-1β, IL-1β; Monocyte Chemoattractant Protein-1, MCP-1 and of c-Jun N-terminal kinase/Activator protein 1, JNK-AP1) activation (Vukic et al., 2009). Platelets are also able to adhere to the vascular wall, leading to sustained platelet recruitment in these plaques and potentially to full vessel occlusion, producing increased platelet activation, increased Aβ_40_ peptide secretion, development of CAA, dementia and, finally acceleration of the progression of AD (Canobbio et al., 2013; Gowert et al., 2014).

Aβ_40_ peptide activates and promotes platelet adhesion and aggregation (Shen et al., 2008; Canobbio et al., 2014), mediated by different receptors such as CD36 and GPIbα, triggering several signal transduction pathways involving p38MAPK, COX1 and synthesis of TXA2, which ultimately increase Ca^2+^ levels, activates calpain and increases Aβ_40_ peptide secretion (Herczenik et al., 2007). The thrombin receptor PAR1 could also have a role in the consequent activation of p38 MAPK and cytosolic phospholipase A2 (PLA2), and TXA2 formation (Shen et al., 2008).

Also Aβ_40_ peptides may modify platelet shape change and granule release through activation of the small GTPase activation of the small GTPase RhoA and phosphorylation of its downstream effector, myosin light chain kinase, involving cytoskeletal reorganization (Canobbio et al., 2014). In platelets Aβ_40_ peptides may also regulate phosphatidylserine exposure, production in platelets is also linked to increased Aβ_40_ levels (Gowert et al., 2014). A correlation between increased ROS formation in AD platelets an increased oxidative stress in AD patients has been demonstrated (Cardoso et al., 2004).

The complex amyloidogenic pathway is also post-transcriptionally regulated by miRNAs at several levels (Table 1 and Supplementary Table S1). Among the validated AD-related targets it is worth mentioning fibrinogen (*FGF*), a clotting protein that contributes to Aβ deposition and is regulated by miR-144-3p, miRNA found in platelet EVs (Cortes-Canteli et al., 2012). By the same token, *BACE1*, the enzyme responsible for β-secretase cleavage of APP, which is regulated by miR-9, miR-29a/b-1, miR-124, miR-195, miR-285, miR-298 among others. Finally, *APP* is also tightly regulated by some platelet-related miRNAs such as let-7i, miR-16, miR-20a, miR-101, miR-106a/b, and miR-155, and by other miRNAs formally miR-17, miR-147, miR-153, miR-323-3p, miR-644, and miR-655 (Maciotta Rolandin et al., 2013). *APP* is therefore fine-tuning regulation by miRNAs through three different means: directly, indirectly, and by regulation of its alternative splicing (Maciotta Rolandin et al., 2013).

Recently platelet activation has been related to an increase in the levels of inflammatory mediators, i.e., Chemokines (RANTES, PF4, MIP-1α), interleukins (IL-1β, IL-7, and IL-8), prostaglandins, CD40L and these proteins can perpetuate platelet activation (Thomas and Storey, 2015). The increased levels of all these inflammatory proteins are associated with AD (Leung et al., 2013). Therefore, uncontrolled platelets’ activation could mediate a chronic inflammatory reaction associated with AD progression, favoring a feed-forward circle that increases inflammation and release of more Aβ_40_ peptides.

During platelet activation the secretion of Aβ peptide considerably increases, for example Kucheryavykh et al. (2017) showed that during clot formation the release of Aβ peptide significantly increased 500 times in the clot formation site. The study also detected Aβ peptide around blood vessels and brain cortex, even determining the presence of Aβ peptide at a very close distance to the entorhinal cortex, this place is the principal zone affected by AD (Selkoe, 1991; Honig et al., 2003). This confirms that the circulating Aβ peptide could be deposited in different brain tissues and contributes to the development of AD. Different mechanisms exist by which the peptide could pass through of the blood–brain barrier BBB: (a) binding to different apolipoprotein such as apolipoprotein J (ApoJ) or clusterin, a heterodimeric glycoprotein that binds Aβ at a binding ratio of 1:1 and with and affinity constants of Kd = 2.0 nM (Shayo et al., 1997), (b) binding to apolipoprotein E (APOE) is a protein with 299 amino acids and transports lipoproteins, fat-soluble vitamins and cholesterol, in the nervous system, astrocytes and microglia (Garai et al., 2014), *APOE* is polymorphic, with three major alleles (*epsilon* 2, *APOE2*; *epsilon* 3, *APOE3*; and *epsilon* 4, *APOE4*). The presence of the *APOE4* is considered a risk factor for AD (Roses and Saunders, 1994). One factor that triggers cell death in the brains of AD patients is oxidative stress and platelets are an important source of oxidative stress (Marcourakis et al., 2008), Marcourakis showed an increase in thiobarbituric acid-reactive substances (TBARS) content and in the activities of Na, K-ATPase and nitric oxide synthase in patients carrying the *APOE4* allele in AD patients (Marcourakis et al., 2008). A decrease in the activity of cytochrome oxidase has been reported in neurons but also in platelets from AD patients (Hirai et al., 2001). Recently, it is reported that the *APOE4* allele inhibits the activity of cytochrome oxidase (Wilkins et al., 2017), confirming the association between APOE alleles and AD risk. Finally, Rosenberg et al. (Rosenberg et al., 1997) showed an altered processing of APP in platelets of AD patients carrying the *APOE4* allele, this alteration may contribute to chronic platelet activation in AD patients. Moreover, these data may relate to alterations in the Amyloid precursor protein processing that may occur in specific areas in the AD brain; (c) binding to RAGE, a multiligand receptor in the immunoglobulin superfamily, Mackic et al. (1998) showed that binding, endocytosis, and transcytosis of Aβ_40_ peptide in brain microvascular was inhibited in 63% by anti-RAGE antibody and the inhibition of RAGE suppresses accumulation of Aβ peptide in brain parenchyma in a mouse transgenic model also showing a reduction in the expression of proinflammatory cytokines, i.e., TNF-α in the brain and production of Endothelin-1 ET-1 (Deane et al., 2003). Binding to the low-density lipoprotein receptor-related protein 1 (LRP1), a multifunctional scavenger and endocytic receptor, a member of the LDL receptor family has been linked to AD and CAA that may regulate Aβ_40_ peptide uptake. LRP1 was demonstrated to be substantially inhibited by anti-LRP-1 antibodies in mice (Deane et al., 2009) and two transporters the ABCB1 and ABCG2 members of the superfamily of ATP-binding cassette ABC transporters, Zhang et al. (2013) found that an injection of Aβ_40_ peptide fluorescent in mice that was quickly cleared of circulation between 30 min and 2 h. and this quickly increased the fluorescence in the brain in KO mice compared with wild type animals. On the other hand, different studies showed decreased expressions of these transporters in elderly people (Wu et al., 2009; Chen et al., 2016).

### Platelets and Neurofibrillar Tangles

One of the principal hallmarks of AD is the presence of neurofibrillary tangles mainly composed of hyperphosphorylated Tau cytoskeletal microtubule-associated protein (Iqbal et al., 2010). Recently this protein has been detected in the platelet proteome and the levels of oligomeric Tau species have been proposed as a novel and robust AD biomarker (Neumann et al., 2011) and correlate with the cognitive status in these patients (Farías et al., 2012) although these results need to be further validated. Platelets also express glycogen synthase kinase 3β (GSK3β), one of the many protein kinases involved in Tau hyperphosphorylation (Forlenza et al., 2011). A complete profile of Tau hyperphosphorylation in platelets may provide fundamental insights into the post-translational modifications of Tau, and may be a surrogate proxy of neuronal dysfunction.

Remarkably, recent findings support a significant role for miRNAs in the regulation of Tau at several levels. These associations include regulation of Tau splicing, and therefore the ratio between different Tau isoforms modulated by miR-132 (Smith et al., 2011), Tau post-transcriptional regulation by miR-219 (Hébert et al., 2010), hyperphosphorylation of the Tau protein through either up-regulation of ERK kinases by miR-15 (Hébert et al., 2010; Santa-Maria et al., 2015) or via activation of the cyclin-dependent kinase 5. The last is associated with over-expression of one the more abundant miRNAs in platelets, miR-26b (Absalon et al., 2013) (Table 1). Finally, miRNAs may also be affecting the clearance of the Tau protein from the circulation through the repression of SIRT1 by miR-9, and by the platelet-related miR-34 and miR-181c (Schonrock and Götz, 2012) (Table 1 and Supplementary Table S1).

In agreement with miRNAs having a role in the pathophysiology of AD, evidence also indicates that miRNAs show abnormal expression in AD. As shown in Table 1 and Supplementary Table S1, the expression levels of several brain-enriched miRNAs miR-9, miR-29a/b, miR-128, miR-134, miR-137, miR-146a, and miR-339 among others and platelet-related miRNAs miR-25, miR-29a/b, miR-30e, miR-34a/c, miR-103, miR-130a, miR-146a, and miR-200c, among others were found to be significantly deregulated in plasma, serum, cerebrospinal fluid and/or brain from AD patients (Schipper et al., 2007; Cogswell et al., 2008; Hébert et al., 2008; Alexandrov et al., 2012; Cheng et al., 2013; Bhatnagar et al., 2014; Burgos et al., 2014; Kumar et al., 2017). Interestingly, some of these miRNAs, such as miR-29 and miR-146a, are found in platelet EVs (Pienimaeki-Roemer et al., 2017) and could take part in the existing intercellular communication between the central nervous and the vascular systems.

## Platelets and Parkinson’s Disease

Parkinson’s disease is clinically characterized by a plethora of symptoms such as resting tremor, bradykinesia, rigidity, and postural imbalance. The pathological explanation underlying these traits is the selective death of dopaminergic neurons located in the substantia nigra (Lotharius and Brundin, 2002).

These neural losses are a consequence of the accumulation of abnormal aggregates of protein alpha synuclein, which is the major structural element in Lewy bodies that develop inside nerve cells (Popescu et al., 2004), but are also a product of the proteasomal system dysfunction, reduced mitochondrial enzymes activities and oxidative stress accompanying aging (Puspita et al., 2017).

Although inflammatory changes are thought to be mainly caused by neuronal destruction and a risk factor for PD, an increased concentration of the same neuroinflammatory markers mentioned above for AD, i.e., RANTES, MIP-1α, IL-1β, TNF-α have even been detected in PD (Reale et al., 2009).

Another characteristic of PD is the increased oxygen consumption and increased ROS production. ROS is produced by platelets under many conditions and can dramatically increase as a consequence of several circumstances such as inflammation. Increase in ROS levels may produce cellular lesions and damage that may eventually lead to cell death (Qiao et al., 2018), it is also is important to mention that ROS is related with platelet hyperactivation and platelet secretion (Carrim et al., 2015) producing a cycle that increases all the previously mentioned components related to neurodegenerative diseases.

According to several reports, changes in the ultrastructure, mitochondrial dysfunction, increased glutamate level and morphology of platelets have been observed in patients with PD (Keane et al., 2011). The mitochondria have a dual function because as they produce and are a ROS target, their deregulation also plays a critical role in PD pathogenesis; this organelle has many functions such as energy generation, calcium homeostasis and response to stress and cell death. Therefore, any damage related to their dysfunction leads to cellular damage and is related to neurodegeneration (Dias et al., 2013).

Mitochondrial dysfunction was first linked to PD upon the recognition that 1-methyl-4-phenyl-1, 2,3,6-tetrahydropyridine (MPTP) induced PD in a study on drug abusers (Perier and Vila, 2012). The MPTP is metabolized into 1-methyl-4-phenylpyridinium MPP+ by the monoamine oxidase B (MAO-B) produced in platelets, crosses the blood-brain barrier and inhibits Complex I of the mitochondrial electron transport chain producing neuronal degeneration (Lim et al., 2009). Monoamine oxidases (MAO) are a family of enzymes belonging to the flavin-containing amine oxidoreductases (Danielczyk et al., 1988) that catalyze the oxidative of monoamines. In the mitochondria they are bound to the outer membrane in most cell types in the body.

Oxygen is frequently used to remove amines from several molecules by the action of MAO producing different groups such as aldehyde and ammonia. The enzymatic capacity of MAO degrades amine neurotransmitters, such as dopamine, norepinephrine, and serotonin (Shih et al., 1999). Two isoforms of MAO, A and B exist, while MAO-A is specialized on the oxidation of serotonin 5-hydroxytryptamine, 5-HT and norepinephrine (NE), MAO-B is specialized on the oxidation of phenylethylamine (PEA) (Shih et al., 1999). There are two forms that can oxidize dopamine (DA). Platelets also possess mitochondrial MAO-B, this enzyme mediates the toxicity of MPTP by catalyzing the formation of the MPP+ which produces PD (Steventon et al., 1989; Götz et al., 1998). In addition, some studies report high dopamine uptake in patients with PD (Roussakis et al., 2015).

miRNAs are crucial in the regulation of redox-signaling pathways associated with several pathological processes related to PD such as mitochondrial dysfunction, α-Synuclein (α-Syn) aggregation, and neuroinflammation (Hsu et al., 2000; Tansey et al., 2007; Subramaniam and Chesselet, 2013; Emde and Hornstein, 2014; Xie and Chen, 2016). As an example, the brain-enriched miRNAs miR-7 and miR-153 have important roles in the regulation of α-Syn expression prompted by mitochondrial ROS-mediated action, both miRNAs can synergistically down-regulate α-Syn expression (Junn et al., 2009; Thompson et al., 2016; Xie and Chen, 2016) and may be associated with the familial form of PD through deregulation of the leucine-rich repeat kinase 2 (*LRRK2*) gene (Gehrke et al., 2010). Of particular interest is miR-34, a brain-enriched geromiR that has been found down-regulated in the amygdala, cerebellum and frontal cortex from PD patients (Miñones-Moyano et al., 2011). In this work miR-34b and miR-34c were demonstrated to alter the mitochondrial function in neuronal cells through the inhibition of protein deglycase (*DJ1*, also known as *PARK7)*, a redox-sensitive protein which triggers activation of antioxidant defenses via the Nrf2/ARE system, and Parkin (*PRKN*), which, together with *PARK7*, are associated with familial forms of PD (Dodson and Guo, 2007). Down-regulation of miR-133b and miR-34b/c was also detected in mid-brain dopaminergic neurons of patients with PD (Kim et al., 2007; Miñones-Moyano et al., 2011). Interestingly, miR-34 had previously been shown to be an enhancer of megakaryocitopoiesis (Gatsiou et al., 2012). Moreover, as shown in Table 1 and Supplementary Table S1, elevated blood expression of the platelet-related miRNAs miR-22-3p, miR-146a and miR-155 were found to be a possible PD-specific miRNA signature (Margis et al., 2011; Caggiu et al., 2018). According to miRNAs as potential biomarkers for PD, one of these studies identified three miRNAs carried by platelet EVs miR-16-2, miR-26a2 and miR-30a as able to differentiate levodopa treated patients compared to untreated patients with PD (Margis et al., 2011). In the same direction, a recent investigation also showed that three other platelet-related miRNAs miR-29a-3p, miR-30b-5p, and miR-103a-3p were over-expressed in PD patients treated with levodopa *versus* untreated PD patients (Serafin et al., 2015).

Potential target genes for these three last miRNAs comprise genes implicated in PD such as, *LRRK2* predicted target for miR-30b-5p and miR-103a-3p; *PARK7* predicted target for miR-29a-3p; the G Protein-Coupled Receptor 37 (*GPR37*) a modulator of the dopaminergic system predicted target for miR-29a-3p; the cell division control protein 42 homolog, *CDC42*, a candidate gene for PD involved in neural death and the antiapoptotic predicted target for miR-29a-3p and miR-103a-3p; and the apoptosis regulator (*BCL2*), predicted target for miR-29a-3p, miR-30b-5p, and miR-103a-3p, which down-regulated by the above stated miRNAs could at least partially responsible for the death of dopaminergic neurons (Serafin et al., 2015).

## Platelets and Multiple Sclerosis

Multiple sclerosis (MS) is a neurodegenerative disease primarily related to damage in the brain and spinal cord CNS, but it may also affect the peripheral nervous system. In this disease the immune system attacks the protective sheath myelin that covers nerve fibbers and causes communication problems between the brain and the rest of the organism (Sheremata et al., 2008). Signs and symptoms depend on nerve damage and affected nerves, the patients may lose the ability to walk independently and cognitive impairment may also appear. There is no known cure for MS. However, treatment can help speed recovery from attacks, modify the state of many diseases and manage symptoms.

The principal damage is characterized by immune-mediated responses with microglial activation and cellular infiltration. These alterations are mainly associated with inflammation of white matter and lead to a progressive demyelination and the destruction of axons (Høglund and Maghazachi, 2014).

Oxidative stress in patients with MS is associated with an increase in myelin and axonal damage that may lead to the apparition of clinical symptoms (Ohl et al., 2016). Different studies related the presence of lesions in MS patients with the apparition of proteins of the coagulation cascade (Morel et al., 2015). There are a lot of ways in which platelets may contribute to the pathophysiology of MS. For example, platelets may modulate inflammation in relation to leukocytes interaction and the release several mediators, i.e., matrix metalloproteinases and chemokines (Sheremata et al., 2008; Horstman et al., 2010). Platelets could be essential for the production of IL1-α, this can activate the endothelium in the brain thus allowing the entry of white blood cells and producing cerebrovascular inflammation, which has a significant role in the production of brain injury in MS. It has been found that the platelets are abundant in the inflamed brain and spinal cord of subjects with MS (Horstman et al., 2010). Under normal conditions the BBB attends to prevent infiltration and adhesion of inflammatory cells into the brain, but the proinflammatory states or damage may allow that different cells to penetrate in the BBB. In this situation the platelets rapidly adhere to the endothelium cells, become activated and secrete bioactive mediators, in this way platelets may contribute to BBB permeability and increases the neurovascular inflammation typical of MS (Morrell et al., 2014).

Platelets may also recognize specific glycolipid structures, i.e., sialated gangliosides in brain, again promoting neuroinflammation and then neurovascular damage. The platelets that penetrate the BBB may recognize sialated gangliosides within the lipid rafts and accumulate, releasing the above stated molecules and so could play an important role in neuronal damage in the induction and perpetuation of inflammation in the CNS (Wachowicz et al., 2016).

Another mechanism whereby platelet are related to CNS damage in MS patients is through the production of ROS (Wachowicz et al., 2016). These levels can dramatically increase under neuroinflammation producing lesions and damage to different cellular structures and potentially cell death. Oligodendrocytes are more sensitive to oxidative damage than astrocytes and microglia. The reactive species may activate the macrophages promoting the damage of the myelin sheath by the attacking to this structure.

In CNS activated platelets may represent an additional source of ROS and therefore could lead to an increase in oxidative stress, which may be at least partially related to the characteristic neuronal demyelination and tissue damage of MS.

Therefore, platelet activation could be a great consequence of the disease, perhaps secondary to an endothelial lesion. Many studies have reported a strong association between MS and the increase in platelet adhesiveness, which would be strongly associated with the activity of the disease. Importantly, platelets contain at least 300 proteins, many of them being involved in the regulation of inflammation, platelets participate in one of the most important pathological processes of MS, mainly a product of the activation of the immune system against the CNS myelin at the beginning.

Moreover, various miRNAs, primarily miR-155 and miR-326, are involved in the regulation of neuroinflammatory processes observed in MS have been associated with disease activity and duration (Zhang et al., 2014; Jagot and Davoust, 2016). Particularly the platelet-enriched geromiR miR-155, which is up-regulated in MS patients, down-regulates CD47 in astrocytes and oligodendrocytes and could contribute to MS-associated inflammation and neurodegeneration. Current reports also show that free circulating miRNAs, including at least four platelet-enriched miRNAs (miR-16, miR-20, miR-22, and miR-145), are deregulated in MS fluids such as plasma, serum, or cerebrospinal fluid (Siegel et al., 2012; Keller et al., 2013; Søndergaard et al., 2013) (Supplementary Table S1). A recent study aimed at the use of exosomal miRNA profiles as signatures in MS identified nine miRNAs as informative biomarkers to distinguish relapsing-remitting from progressive MS (Table 1), which includes two platelet-enriched miRNAs, miR-30b-5p and one of the major miRNA drivers of platelet production, miR-223 (Ebrahimkhani et al., 2017). Interestingly, miR-223 has also been identified as a potential MS biomarker across several independent blood-based miRNA studies (Fenoglio et al., 2016) and has been involved in the pathophysiology of MS by targeting the transcription factor *STAT5* and other inflammatory regulators implicated in MS such as heat shock protein 90 and E2 (Ebrahimkhani et al., 2017). In the same study other miRNAs were also found as promising candidate biomarkers for relapsing-remitting MS and progressive MS, including the platelet-related miRNAs miR-30b-5p, miR-223, and miR-15-5p, the last predicted as regulator of the fibroblast growth factor-2 gene (*FGF2*), a gene involved in demyelination and remyelination (Fenoglio et al., 2012).

## Platelets and Other Neurodegenerative Diseases

Huntington’s disease (HD) is a neurodegenerative genetic disorder originated by the expansion of the single tandem repeat CAG in the Hungtingtin gene (HTT) and it is characterized by the occurrence of abnormal involuntary movements, cognitive decline and psychiatric disorders such as depression (Arrasate and Finkbeiner, 2012).

The finding of hyperactive platelets is the principal characteristic in HD patients in response to many agonists such as epinephrine, dopamine, serotonin, adenosine diphosphate, arachidonic acid, and collagen (Muramatsu et al., 1982). This characteristic can be explained as the levels of NO are very diminished in platelets in HD patients, it is important to note that NO is a potent vasodilator and inhibitor of platelet activation (Carrizzo et al., 2014). Under this state of platelet hyperactivity there is an increase in the inflammatory components secreted by platelets that allow amplifying the proinflammatory state in patients with HD, a component that is related to HD development and prognostics.

Mutant Huntingtin represses the formation of P bodies through its interaction with Ago1 and Ago2, two proteins that are crucial for the biogenesis of miRNAs (Savas et al., 2008). Thus, deregulation of several miRNAs, miR-9, miR-16, miR-22, miR-29a/b, miR-132, miR-196, and miR-330 among others, has been reported in the brain of HD patients (Johnson et al., 2008; Packer et al., 2008; Martí et al., 2010). Efforts are nowadays focusing on identifying miRNAs whose expression in the blood could correlate with disease progression as is the case of the platelet-related miR-34b that is regarded as a reliable and promising HD biomarker prior to the beginning of symptoms (Gaughwin et al., 2011) and the case of the platelet-enriched miRNAs miR-22-5p, miR-30d-5p, and miR-223 (Díez-Planelles et al., 2016). Furthermore, a therapeutic role for some miRNAs, the most remarkable being miR-27 and miR-196a, has been suggested in HD (Packer et al., 2008; Maciotta Rolandin et al., 2013; Ban et al., 2017).

Another biological component that has been used as biomarkers is MAO. Some studies relate the neuronal damage with the high levels of MAO-A and MAO-B activity in brain and platelets and the HD progression (Markianos et al., 2004). The expression of this protein is regulated by the transcription factors in response to stress such as ischemia and inflammation (Gupta et al., 2015).

An increase in the mitochondrial-dependent apoptosis in platelets in HD patients has also been shown. Based on this evidence, it is justified that, on the whole, platelets play a significant role in HD (Ehinger et al., 2016).

Amyotrophic lateral sclerosis (ALS) is a disease characterized by a gradual degeneration of motor neurons and neuromuscular paralytic disorder that leads to respiratory failure and death (Kiernan et al., 2011). The main etiological factors responsible for ALS are found in the CNS and in peripheral tissues, for example skeletal muscle, liver, lymphocytes, platelets etc. In this sense, mitochondrial dysfunction and changes in the ultrastructure of ALS platelets such as alterations in permeability transition and in mitochondrial membrane potential (MMP) have been found related to ALS (Shrivastava et al., 2011).

Increased glutamine synthetase together with normal expression of excitatory amino acid transporter 2 responsible for over 90% of glutamate reuptake within the CNS in the platelets of ALS patients, involving glutamate excitotoxicity in the pathogenesis of ALS has also been reported (Bos et al., 2006). A significant decrease of serotonin, a molecule that controls motor neuron excitability and energy metabolism, has also been observed in the platelets of ALS patients (Dupuis et al., 2010). On the other hand, thrombospondin, a glycoprotein released from platelet alpha-granules, has found significantly increased in ALS patients suggesting stressing the potential of platelets as biological markers.

As for the previous neurodegenerative diseases, miRNAs also show up as promising biomarkers for ALS. Profiling of miRNAs in ALS patients has been performed including analysis of blood samples and peripheral tissues (Butovsky et al., 2012; De Felice et al., 2012; Campos-Melo et al., 2013; Koval et al., 2013; Takahashi et al., 2015). Among these studies Butovsky identified an inflammatory miRNA signature in CD14+CD16- monocytes from ALS patients. Deregulated miRNAs in ALS monocytes include miR-338-3p, a miRNA that has also been found significantly deregulated in blood, cerebrospinal fluid, serum, spinal cord, and brain from ALS patients (Shioya et al., 2010; De Felice et al., 2012). This last study identified, for the first time, specific disease-related changes in miRNAs as putative biomarkers for early diagnosis of the ALS. Differential miRNA expression in the spinal cord of ALS patients leads to the identification of up-regulation of two platelet-related miRNAs, miR-146 and miR-155, in ALS patients compared to healthy controls (Campos-Melo et al., 2013; Shaner et al., 2013) (Table 1). These two miRNAs are considered geromiRs and have been largely associated with inflammatory processes (Olivieri et al., 2013) with miR-155 being considered a promising therapeutic target for ALS. Moreover, miR-146 has also been reported to directly regulate the low MW neurofilament (*NFL*) mRNA, which may points toward the involvement of miR-146 in the repression of *NFL* observed in the spinal motor neurons of ALS patients (Campos-Melo et al., 2013).

## Conclusion

From the elements of human blood, platelets are about one of the most important derivatives from megakaryocytes in the bone marrow. Platelets are crucial in the regulation of thrombosis and hemostasis as well as in vessel constriction, repair and clot retraction. In addition to their haemostatic function, platelets have an essential role during inflammatory processes and are an important source of proinflammatory molecules such as P-selectin, tissue factor, CD40L and metalloproteinase.

Overall, biochemical alterations in patients suffering from neurodegenerative disorders may not only occur in the brain, but also could affect blood vessels and blood cells. In this regard, vascular and metabolic disorders associated with aging are recognized risk factors for neurodegeneration.

Extracellular vesicles secreted by platelets may function as intercellular communicators, carrying pathologic neurological disease-related molecules, such as miRNAs, from the circulation into other organs and tissues such as the brain, reinforcing the existence of a molecular association between vascular and neurodegenerative disorders. It is worth noting that miR-34a/b/c, miR-146, and miR-155, three miRNAs with important roles in platelet production and function, have also been found to be repeatedly involved in the regulation of neurodegeneration-related processes and are being considered as potential geromiR biomarkers for numerous neurodegenerative disorders. According to this, platelets could represent a major source and vehicle of miRNAs, e.g., miR-223 that serve as secreted molecules acting on target cells other than the ones where they are produced.

**Figure 1:**
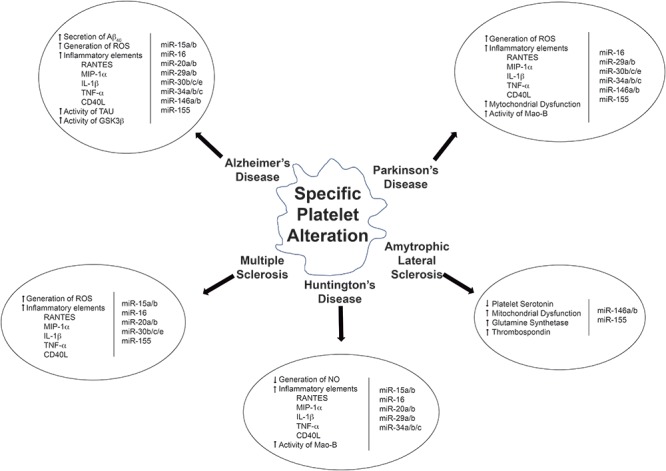
Overview of the relationship between altered molecular pathways and deregulated miRNAs in platelets.

Activated platelets are crucial in the development of major diseases like CNS diseases AD, PD, MS and others and also as potential biomarkers for neurological diseases, they are easy to obtain, manipulate and analyze (Figure 1). Platelets seem to be important executors of neuropathological diseases and therefore platelets might be regarded as novel therapeutic targets for neurodegeneration. Platelets could somehow reflect what is happening in the CNS along the course of neurodegenerative pathological states, and therefore could be promising biomarkers for early onset diagnosis of a pathological condition. Different from neurons, platelets are easy to work with and could be thus considered as a promising and effective tool on the study neurodegeneration. Therefore, platelets and their secreted molecules, including EVs and platelet miRNAs, remain as promising peripheral biomarkers in understanding the diagnosis and prognosis of neurodegenerative disorders.

## Author Contributions

YE-P, CG-B, EF, and IP wrote the original draft of the manuscript. YE-P and MA wrote, reviewed, and edited the manuscript.

## Conflict of Interest Statement

The authors declare that the research was conducted in the absence of any commercial or financial relationships that could be construed as a potential conflict of interest.
