# Supplementary material for: Decoding the Role of Platelets and Related MicroRNAs in Aging and Neurodegenerative Disorders
Source: Front Aging Neurosci. 2019 Jul 2;11:151. doi: 10.3389/fnagi.2019.00151 (PMC6614495; doi:10.3389/fnagi.2019.00151)
Supplement: Supplementary file 1 [file Table_1.docx]

**Supplementary Table S1. Platelet-related miRNAs reported to be involved in neurodegenerative disorders**

| **miRNA** | **Disorder** | **Evidence of miRNA involvement in the disorder** | **References** |
| --- | --- | --- | --- |
| let-7f 4,5 | AD | Up-regulated in the cerebrospinal fluid of patients | Cogswell et al., 2008 |
| let-7i 1,4,5 | AD | Regulation of the amyloid precursor protein *APP* experimentally validated target | Maciotta Rolandin et al., 2013 |
| miR-101-3p 5 | AD | Regulation of *APP* experimentally validated target | Vilardo et al., 2010 |
| miR-103 1,3,4 | PD | Up-regulated in the blood of levodopa treated patients potential PD-specific miRNA signature. Predicted Regulation of *LRRK2*, *CDC42* and *BCL2* | Serafin et al., 2015 |
| miR-106b 5 | AD | Regulation of *APP* experimentally validated target | Maciotta Rolandin et al., 2013 |
|  | HD | Up-regulated in the frontal cortex of patients | Martí et al., 2010 |
| miR-107 5 | AD | Down-regulated in the brain of patients. Regulation of the β-secretase *BACE1* experimentally validated target | Wang et al., 2008 |
| miR-126 1,2,4,5 | AD | Down-regulated in the cerebrospinal fluid of patients | Cogswell et al., 2008 |
| miR-130a 3 | AD | Up-regulated in the peripheral blood mononuclear cells of patients | Plé et al., 2012 |
|  | HD | Up-regulated in the plasma of patients | Díez-Planelles et al., 2016 |
| miR-144-3p 5 | AD | Regulation of fibrinogen *FGF* experimentally validated target | Cortes-Canteli et al., 2012 |
| miR-145-5p 5 | MS | Deregulated in the blood of patients | Søndergaard et al., 2013 |
| miR-146a 2,5 | AD | Up-regulated in the hippocampus of patients | Müller et al., 2014 |
|  | AD | Deregulated in several areas of the brain of patients | Cogswell et al., 2008 |
|  | AD | Up-regulated in the frontal cortex of patients | Swarbrick et al., 2019 |
|  | AD | Down-regulated in plasma of patients | Kiko et al., 2014 |
|  | AD | Up-regulated in cerebrospinal fluid of patients | Alexandrov et al., 2012 |
|  | AD | Regulation of the complement factor H *CFH* and the Toll-like receptor 4 *TLR4* | Pienimaeki‐Roemer et al., 2017 |
|  | PD | Down-regulated in the blood of patients potential PD-specific miRNA signature | Caggiu et al., 2018 |
|  | ALS | Up-regulated in the spinal cord of patients. Regulation of the low MW neurofilament *NFL* | De Felice et al., 2012 |
|  | ALS | Up-regulated in CD14+ CD16− monocytes of patients | Butovsky et al., 2012 |
| miR-146b 5 | ALS | Up-regulated in the spinal cord of patients. | Koval et al., 2013 |
|  | AD | Down-regulated in cerebrospinal fluid of patients | Cogswell et al., 2008 |
| miR-155 2 | AD | Regulation of APP | Maciotta Rolandin et al., 2013 |
|  | AD | Up-regulated in cerebrospinal fluid of patients | Alexandrov et al., 2012 |
|  | PD | Up-regulated in blood of patients. Good candidate as a disease progression biomarker. Promising as target for anti-inflammatory therapy | Caggiu et al., 2018 |
|  | MS | Up-regulated in the brain and plasma of patients. Regulation of *CD47* in astrocytes and oligodedrocytes | Jagot and Davoust, 2016 |
|  | ALS | Up-regulated in the spinal cord of patients. Potential therapeutic target. | Koval et al., 2013 |
|  | ALS | Up-regulated in CD14+ CD16− monocytes of patients | Butovsky et al., 2012 |
| miR-15a/b 2,5 | AD | Hyperphosphorylation of Tau protein through up-regulation of ERK kinases | Hébert et al., 2010 |
|  | AD | Down-regulated in the plasma of patients | Wang et al., 2011 |
|  | MS | Potential informative biomarker to distinguish relapsing-remitting from progressive MS | Ebrahimkhani et al., 2017 |
|  | MS | Predicted regulation of he fibroblast growth factor-2 and Kinesin family member 1B | Fenoglio et al., 2012 |
|  | HD | Up-regulated in the frontal cortex of patients | Martí et al., 2010 |
| miR-16 2,5 | AD | Regulation of APP | Maciotta Rolandin et al., 2013 |
|  | PD | Up-regulated in the blood of levodopa treated patients | Margis et al., 2011 |
|  | MS | Up-regulated in the blood of patients | Keller et al., 2014 |
|  | HD | Up-regulated in the striatum and frontal cortex of HD patients | Martí et al., 2010 |
| miR-181a-5p 5 | AD | Affecting the clearance of Tau from the circulation through repressing the sirtuin *SIRT1* | Schonrock and Götz, 2012 |
| miR-200c 2 | AD | Deregulated in several areas of the brain of patients | Cogswell et al., 2008 |
|  | AD | Up-regulated in the plasma of patients | Wu et al., 2016 |
| miR-20a/b | AD | Regulation of APP | Hébert et al., 2009;Maciotta Rolandin et al., 2013 |
|  | MS | Down-regulated in the blood of patients | Keller et al., 2014 |
|  | HD | Up-regulated in the frontal cortex of patients | Martí et al., 2010 |
|  | MS | Up-regulated in the plasma of patients potential MS biomarker | Siegel et al., 2012 |
| miR-22 5 | PD | Down-regulated in the blood of patients potential PD biomarker | Margis et al., 2011 |
|  | HD | Up-regulated in the plasma of HD patients | Díez-Planelles et al., 2016 |
|  | HD | Down-regulated in the Brodmann’s area 4 of patients | Packer et al., 2008 |
| miR-223 1, 2, 4 | MS | Potential informative biomarker to distinguish relapsing-remitting from progressive MS. Regulation of the transcription factor STAT5 | Ebrahimkhani et al., 2017 |
|  | HD | Up-regulated in the plasma of patients | Díez-Planelles et al., 2016 |
| miR-25 3,4 | AD | Down-regulated in the peripheral blood monocuclear cells of patients | Plé et al., 2012 |
| miR-26a 1,4,5 | PD | Up-regulated in the blood of levodopa treated patients | Margis et al., 2011 |
| miR-26b 1,4,5 | AD | Hyperphosphorylation of Tau protein through activation of the cyclin-dependent kinase 5 | Absalon et al., 2013 |
|  | AD | Up-regulated in the brain and down-regulated in the blood of patients | Swarbrick et al., 2019 |
| miR-27a 5 | ALS | Up-regulated in CD14+ CD16− monocytes of patients | Butovsky et al., 2012 |
| miR-27b 5 | HD | Up-regulated in the striatum and frontal cortex of patients | Martí et al., 2010 |
| miR-29a/b 5 | AD | Degulated in the brain of patients | Hébert et al., 2008 |
|  | PD | Down-regulated in the blood of patients potential PD biomarker. | Margis et al., 2011 |
|  | PD | Up-regulated in the blood of levodopa treated patients. Predicted regulation of *PARK7*, *GPR37*, *CDC42*, *BACE1*, and *BCL2* | Maciotta Rolandin et al., 2013;Serafin et al., 2015 |
|  | HD | Up-regulated in the Brodmann’s area 4 of patients | Johnson et al., 2008 |
|  | HD | Down-regulated in the Brodmann’s area 4 of HD patients | Packer et al., 2008 |
| miR-30b 1, 4 | PD | Up-regulated in the blood of levodopa treated patients potential PD-specific miRNA signature. Predicted targets include *LRRK2* and *BCL2* | Serafin et al., 2015 |
|  | AD | Up-regulated in the cerebrospinal fluid of patients | Cogswell et al., 2008 |
|  | MS | Potential informative biomarker to distinguish relapsing-remitting from progressive MS | Ebrahimkhani et al., 2017 |
| miR-30c 1 | PD | Up-regulated in the blood of levodopa treated patients | Margis et al., 2011 |
| miR-30e 1 | AD | Up-regulated in circulating exosomes of patients | Tan et al., 2014;Cheng et al., 2015 |
|  | AD | Deregulated in several areas of the brain of patients | Cogswell et al., 2008 |
| miR-320 1 | AD | Down-regulatedin the temporal cortex of patients | Swarbrick et al., 2019 |
| miR-34a/b/c 2 | AD | Up-regulated in the frontal cortex of patients | Swarbrick et al., 2019 |
|  | AD | Up-regulated in the hippocampus of patients | Sarkar et al., 2016 |
|  | AD | Deregulated in several areas of the brain of patients | Cogswell et al., 2008 |
|  | AD | Up-regulated in the serum of patients | Bhatnagar et al., 2014 |
|  | AD | Up-regulated in blood monocuclear cells of patients | Burgos et al., 2014 |
|  | AD | Up-regulated in the hippocampus of patients | Müller et al., 2014 |
|  | AD | Affecting the clearance of Tau from the circulation through repressing *SIRT1* | Schonrock and Götz, 2012 |
|  | PD | Down-regulated in amygdala, frontal cortex and cerebellum of PD patients and inhibition of DJ1 *PARK7* and Parkin *PRKN* | Miñones-Moyano et al., 2011 |
|  | HD | Up-regulation in plasma of patients Potential biomarker of HD prior to symptom onset | Gaughwin et al., 2011 |
| miR-486-5p 5 | HD | Up-regulated in the frontal cortex of patients | Martí et al., 2010 |
| miR-92a 5 | HD | Up-regulated in the frontal cortex of patients | Martí et al., 2010 |

Platelet-related miRNAs according to Landry et al., 2009 1; Edelstein and Bray, 2011 2; Plé et al., 2012 3; Simon et al., 2014 4

Platelet-Evs-related miRNAs according to Pienimaeki-Roemer et al., 2017 5
